# Trends of Rural/Urban Homicide in Colombia, 1992-2015: Internal Armed Conflict and Hints for Postconflict

**DOI:** 10.1155/2018/6120909

**Published:** 2018-10-01

**Authors:** Katherine Vallejo, Jose Tapias, Ivan Arroyave

**Affiliations:** ^1^Faculty of Psychology, CES University, Medellin, Colombia; ^2^Faculty of Economic Sciences, Department of Economy, University of Antioquia, Medellin, Colombia; ^3^National School of Public Health, University of Antioquia, Medellin, Colombia

## Abstract

**Objective:**

To analyze the relationship between rural and urban homicide rates in Colombia between 1992 and 2015 and the fluctuations in these rates.

**Methods:**

Individual records of homicides and population aggregates in men and women aged 15-64 years were used. The adjusted rates of annual homicides were calculated for urban/rural areas and standardized by age. Rate Ratios (RRs) adjusted by region were calculated. A joinpoint analysis was performed to identify inflection points and the Annual Percentage Change (APC).

**Results:**

Four joinpoints were identified in rural and urban rates: after peaking in 1992, homicide rates fell until 1997, and then increased until 2002. From this point on there was a continuous reduction until 2015, although this reduction slowed down from 2005 onward. During almost the whole period, the rates of rural homicides were higher than those of urban homicides, although they equalized at the end of the period.

**Conclusions:**

Unlike in other countries, during the study period Colombian homicide rates, which coincided with the dynamics of the armed conflict, were higher in rural than in urban areas. In recent years, a predominance of urban homicides committed by younger men has been identified, which could pose a challenge to postconflict in Colombia.

## 1. Introduction

Colombia is now tackling the possibility of a negotiated peace including several armed actors following seven decades of internal war. The second half of the last century began in Colombia with an especially ferocious period so-called simply “La Violencia”. While political parties involved tempered the partisan violence by negotiating the sharing-alternation of power at 60s, an internal peasants' armed conflict emerged in parallel and disseminated over the ensuing decades. Primary actors were insurgent-revolutionary movements fighting against state armed forces in search of seize power. However, since 80s the conflict became increasingly complex and diverse in terms of actors and interactions: subversion and new forms of illegal counterinsurgency found rents with the irruption of drug trafficking which in turn had its own mechanisms of control and violence. [1]. Therefore, from the early 80s, when the rise of drug trafficking and crime acted as triggers for violence in Colombia [2], there was a constant increase in homicides, with the highest rates in 1992, 1998, and 2002 [3]. Conversely, during the first decade of the 21st century, homicide rate in Colombia decreased by more than half, going from 70 per 100,000 at the beginning of 2000s to 28 per 100,000 in 2010 [4]. Despite this reduction, in 2011 the homicide rate in Colombia was almost twice the average of the other Latin American countries (with which it shares a common history, equally limited social conditions [5], and similar health and social protection systems [6]), almost seven times that of the USA, and 31 times that of Western Europe [7]. This demonstrates the need for the phenomenon of lethal violence in Colombia to be intensively studied so that public policy options can be formulated in order to accelerate its reduction.

Several investigations have agreed on the need for a multidimensional approach to understanding violence and homicide in Colombia, addressing such factors as the effect of social inequalities, the role of institutional weaknesses, and the strong influence of illegal activities [8, 9]. One study argues that in Colombia, unlike in other countries, institutional weaknesses contribute more to high rates of homicide than social inequalities do [10], causing a dynamic of violent and common crime which is only loosely related to social conditions [11]. In Colombia, it has been found that homicides and illicit activities, such as illegal drug markets [2, 7, 12, 13] and illegal groups participating in the armed conflict [7, 13–15], and the availability of firearms (which varies in different parts of the country) [12] are closely related. There is also an inverse relationship with variables associated with the state's capacity of deterrence, such as the number of agents of the armed forces [16, 17], the effectiveness of the judicial system [7, 13], arrests [16], or the territorial presence of the army [7]. Moreover, recent studies have identified the illegal armed groups and their strategic objectives as the main factors in explaining the regional variations in homicide rates in Colombia, which are the result of dissemination mechanisms such as geographic diffusion and relocation of violence [12, 18], and not as a consequence of social inequalities or institutional weaknesses, as previously thought. Regarding the geographical distribution, it has been established that homicide rates are, globally, invariably significantly higher in urban than in rural areas [7, 19]. In the case of Colombia, in the period 1990 to 2005, a spatial analysis reported that these rates were similar in both rural and urban areas. The analysis also evidenced a high geographic concentration of violence, identifying that 64% of homicides occurred in municipalities which contained only 37% of the country's population [18]. Another study for the 1990-2000 period conducted a differential analysis of the behaviour of homicide rates in rural and urban regions according to coca production and showed that rural homicide rates surpassed urban rates in departments where illicit crop growth was widespread, evidencing that it is the civil conflict, as opposed to criminal activity, that is the main cause of the increase in rural violence [14].

Although the effects of the conflict were more pronounced in rural areas, and the increases in the homicide rates coincided with increases in the intensity of armed confrontations [18], the behaviour of the rural and urban tendencies of homicide over an extended period of time—where there have been important changes in the mechanics of the conflict, socioeconomic conditions and the state's capacity for deterrence—remains unknown. This study aims to identify rural and urban fluctuations in homicide rates in Colombia for the period 1992-2015, to pinpoint the moments of high and low intensity, and to explore the possible relationship with the country's armed conflict in light of the historical context. These results could offer inputs to providing public policy recommendations regarding the targeting of efforts in rural and urban contexts with a view to the postconflict period in Colombia, which is becoming more important after the successive peace negotiations in the last decades in Colombia, and whose most important achievement was the agreement reached in 2016 between the state and the oldest and largest guerrilla group in the country [20].

## 2. Methodology

### 2.1. Study Design and Target Population

An ecological study of time trends of rural and urban homicide differences in Colombia between 1992 and 2015 was conducted. To calculate homicide rates by sex, five-year age group, and rural and urban areas, secondary data was used: the individual anonymized database of deaths and the aggregated population estimates based on censuses and surveys. This data is provided by the Colombian National Administrative Department of Statistics (DANE) [21]. The study was limited to young and adult population (between 15 and 64 years of age). For the study period (1992-2015) there were 511,335 homicides, 93.7% of them between 15 and 64 years of age (479,283), which are the focus of this work.

### 2.2. Variables of Interest

The variables of sex, age of death in five-year groups, and region of occurrence of the event were used, disaggregated according to their urban or rural geographic location.

### 2.3. Setting

The official records of homicides in Colombia are compiled from death certificates, as they are universally applicable medical documents designed in accordance with international standards that allow the characterization of mortality according to different variables, including demographics [22]. To identify homicides, codes X85-Y09 and Y87 were used according to the International Classification of Diseases [23]. The period of study was limited by the availability of the variables in the mortality database, since in Colombia information from official sources on causes of death in rural and urban areas is only available from 1992 until 2015, and data were sampled annually from available records for this period.

The location of the homicides was categorized as urban or rural in accordance with the definitions established in the death records and in the population data used: urban areas include municipal seats (where the city halls are located), and rural areas include (i) populated centers (concentrations of buildings corresponding of 20 or more contiguous dwellings) and (ii) dispersed rural areas outside the urban perimeters of the municipal seats, as characterized by the dispersed layouts of dwellings and farms, and unmapped areas. The rural areas do not usually have public services or other facilities typically found in urban areas [24].

### 2.4. Data Analyses

For the purposes of this study, all cases with at least one missing value for the variables of interest were eliminated; therefore 3,643 cases (0.8% of the total) were discarded, leaving 475,640 homicides for the analysis.

Initially, standardized homicide rates were obtained from the World Health Organization (WHO) World Standard Population for 1997 [25]. In the second stage, Rate Ratios (RR) of homicide were calculated by applying Poisson regression models with homicide rates as a response variable and a dummy variable for geographic location (rural and urban) as an explanatory variable, adjusted by sex, five-year age group, and region. The homicide RRs were calculated using the urban area as a reference, so a value greater than 1 means that there is a higher homicide rate in the rural area than in the urban area, whereas a value lower than unity implies a higher frequency of occurrence of lethal violence in urban areas. The analyses were conducted with SAS® 9.4.

Finally, and in order to identify patterns of change in the urban-rural differences in homicide, the public domain software Joinpoint Trend Analysis Software® [26] was used, which has been applied in numerous investigations since its implementation in 2000 [27]. This software takes the trend data (in the case of the present study, the annual standardized rates of homicide and the RR, both with their respective standard of error), uses a Monte-Carlo permutation, and aligns it to the simplest inflection point model possible, in turn describing the Annual Percentage Change with its statistical significance in the intervals between the inflection points.

### 2.5. Study Limitations

Homicide data come from mortality records, while data on the population distribution by rural and urban area come from censuses and demographic projections. This may have led to the so-called numerator/denominator bias, which generally results in an overestimation of disparities [28]

Another limitation is the underreporting of deaths in some regions, which is very common in the poorest and most rural regions [29, 30]. The results, therefore, are indicative of potentially greater differences in rural homicide compared to urban homicide. On the other hand, underregistration according to these same studies has been reducing over time, so the underestimation of trends in rural/urban differences would be reduced over time and the conclusions of the study would not change essentially.

Finally, it is important to note that the limited availability of variables disaggregated by rural and urban areas in Colombia makes it difficult to establish correlations between homicide rates and other conflict variables in small geographic areas and over long periods. Another important limitation is that the registries do not allow the identification of areas where there are more frequent homicides than those where they occur rarely [18], constituting one of the main constraints to unraveling the implications of the phenomenon described in this study.

### 2.6. Ethical Considerations

This study is based on reports of anonymized individual causes of death and on a secondary analysis of aggregated population data, information which is publicly provided by DANE. The research was approved by the Ethics Committee of CES University School of Medicine as it corresponds to the work of KV's master's degree, who was advised by IA. The project on which this research is framed in which IA is IP (see Acknowledgments) was approved by the Ethics Committee of the National School of Public Health of the University of Antioquia. In both instances, the project was rated as “without risk”, according to the regulations in Colombia on the matter.

## 3. Results

Table 1 shows that in Colombia (1992-2015) almost three-quarters of adult homicides (15-64 years of age), of both men (330,602) and women (25,953), occurred in urban areas. 92.6% of adult homicide victims were men, which means that there were 12.5 times more male homicide victims than female ones. The age-standardized homicide rates were higher in rural areas: 107.06 men homicides victims per 100,000 inhabitants in rural areas compared to 91.49 in urban areas. For women, the rate was 9.78 and 6.72, respectively. This data illustrates that the homicide rate in rural Colombia compared to urban areas was 1.17 times higher in men and 1.47 times higher in women. By age groups, young adults (25-44 years) accounted for slightly more than half of the homicide victims, both in men and in adult women, while a third of the victims were between and 15 and 24 years of age.

**Figure 1:**
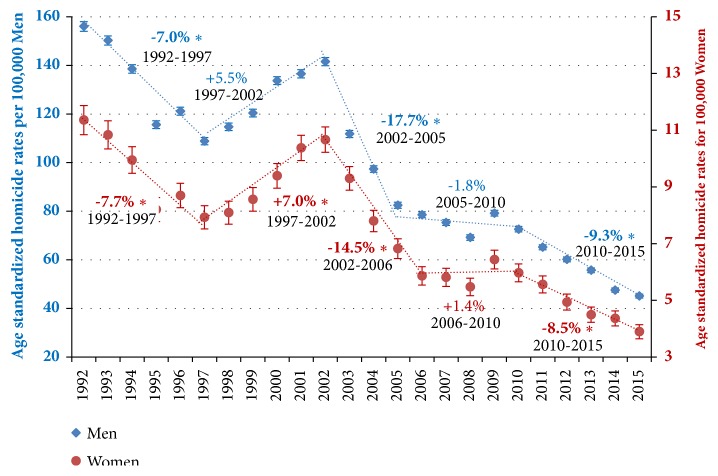
**Trends in age-standardized homicide rates (ASHR) separately for men and women aged 15-64, with 95**%** confidence intervals, including annual percent change (APC) based on joinpoint models, Colombia, 1992-2015.*** Markers: Observed age-standardized homicide rates (ASHR) among men and women of 15-64 years including 95% CI (vertical lines). The points represent ASHR, and the dotted lines represent the trendlines between joinpoints. The numbers adjacent to the lines represent annual percent change (APC) during the corresponding periods (specified below APC values), based on joinpoint modelling; a star in APC indicates statistical significance at α 0.05.*

**Figure 2:**
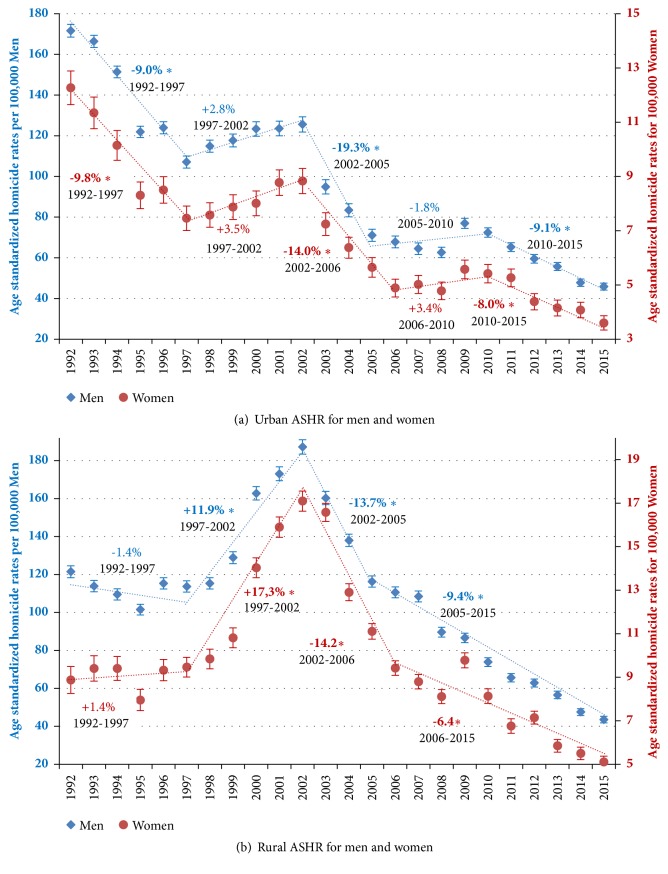
**Trends in age-standardized homicide rates (ASHR) separately for men and women aged 15-64, with 95**%** confidence intervals, including annual percent change (APC) based on joinpoint models, separately for urban and rural area, Colombia, 1992-2015.*** Markers: Observed age-standardized homicide rates (ASHR) among men and women of 15-64 years including 95% CI (vertical lines). The points represent ASHR, and the dotted lines represent the trendlines between joinpoints. The numbers adjacent to the lines represent annual percent change (APC) during the corresponding periods (specified below APC values), based on joinpoint modelling; a star in APC indicates statistical significance at α 0.05.*

Regarding the fluctuations by sex, homicide rates followed very similar trends for both men and women (Figure 1), with four joinpoints that give rise to five well-defined intervals: (i) a significant fall between 1992 and 1997 for men (APC=-7.0%) and women (APC=-7.7%), (ii) a significant increase in 1997-2002 in men (APC=+5.5%) and women (APC=+7.0%), (iii) a pronounced and significant decrease in 2002-2005 in men (APC=-17.7%) and 2002-2006 in women (APC=-14.5%), (iv) followed by a period of stagnation until 2010, (iv) and from that year on a less pronounced, but constant, fall until the end of the period for men (APC=-7.0%) and women (APC=-7.7%). Exactly the same intervals are observed when the fluctuations in urban areas for both sexes are evaluated (Figure 2(a)), although with more pronounced reductions. The urban homicide rates reduced in 1992-2015 by 73% in men and 71% in women, a slightly higher reduction than that found in total for men and women (71% and 66%, respectively).

On the other hand, men and women homicide rates in rural areas (Figure 2(b)) show a similar pattern, identifying four intervals: (i) from the beginning of the period until 1997 there were no significant changes. From this inflexion point all the fluctuations are significant: (ii) an increase in homicides of both sexes until 2002 and a subsequent reduction until the end of the period that occurs in two phases, (iii) a more pronounced reduction from 2002 to 2005 in men and in 2006 in women, (iv) and from this joinpoint the reduction slows down. Rural homicide rates reduced by 77% in men and 70% in women from their peak in 2002 to 2015.

Figure 4 shows how trends in homicide rates evolved separately by sex and areal urban area (four panels) in order to better detail and improve the understanding of the findings described above.

**Figure 3:**
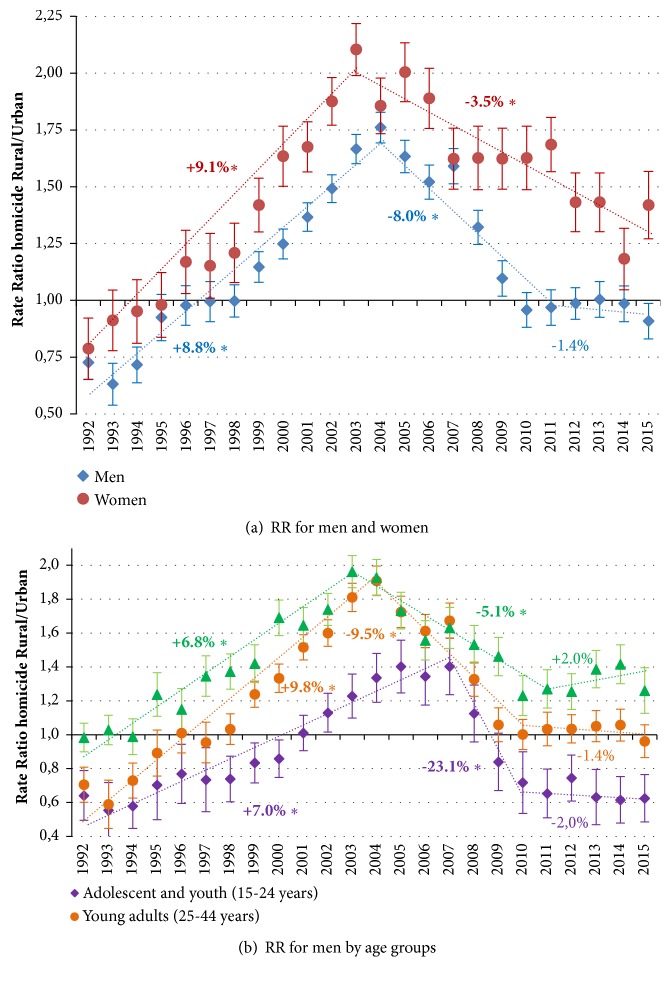
**Trends of Rate Ratio (RR) of rural/urban (reference) age-standardized homicide rates (ASHR) separately for men and women aged 15-64 (and separately by age groups for men), with 95**%** confidence intervals, including annual percent change (APC) based on Joinpoint models, Colombia, 1992-2015.*** Markers: Observed age-standardized homicide rates (ASHR) among men and women of 15-64 years including 95% CI (vertical lines). The points represent ASHR, and the dotted lines represent the trendlines between joinpoints. The numbers adjacent to the lines represent annual percent change (APC) during the corresponding periods (specified below APC values), based on joinpoint modelling; a star in APC indicates statistical significance at α 0.05.*

**Figure 4:**
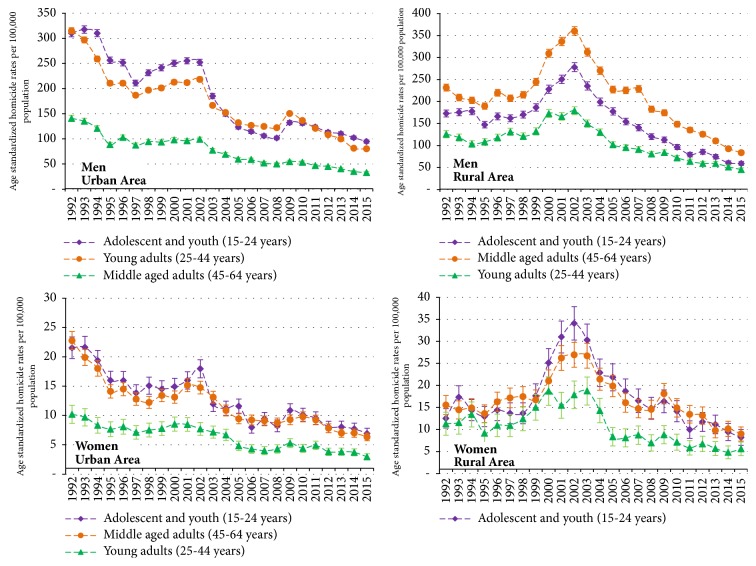
**Trends in age-standardized homicide rates (ASHR) separately for men and women aged 15-64, with 95**%** confidence intervals, including smoothed lines between years, separately for urban and rural area by age groups (adolescent and youth, young adults, and middle aged adults), Colombia, 1992-2015.*** Markers: Observed age-standardized homicide rates (ASHR) among men and women of 15-64 years including 95% CI (vertical lines). The points represent ASHR, and the dotted lines represent smoothed trendlines.*

Figure 3(a) shows the Rate Ratio (RR) comparing the adjusted rates of homicides in urban areas as a reference. For men and women, the RR began in 1992 with observed values lower than 1, indicating more urban than rural homicide rates, but with a progressive tendency in the opposite direction, which extends to 2004 in men (RR=1.8, with APC=+8.8%) and 2003 in women (RR=2.1, with APC=+9.1%), showing that for both sexes homicide rates became progressively higher in rural areas compared to urban areas. From this point, the RR shows a significant and sustained reduction in men until 2011 and in women until the end of the period. It is noted that since 1996, homicide rates in women have been significantly higher in rural than in urban areas, while in men this only occurs between 1999 and 2009. In fact, Table 1 shows that in all groups by age and sex there is a predominance of rural over urban homicides except in adolescent mens. To explain this difference, a disaggregated analysis by age groups was carried out (Figure 3(b)), and it was found that the RR for young men marks this trend. The rural homicide rates were significantly higher only for that population group and only in the short period 2002-2007, while for men over 25 years this behaviour was present for almost the entire period.

## 4. Discussion

Unlike previous consistent evidence from around the world demonstrating a greater burden of urban violence [19], the results of this investigation show that, except for the first and last years of the analyzed period, homicide rates in Colombia were higher in rural areas. As confirmed in previous studies, this phenomenon can be closely related to the armed conflict in Colombia [31], particularly the activity of the illegal armed groups, showing that there is a correlation between the spatial presence of these groups and the high homicide rates, especially in noncombatant civilians in the municipalities [19]. In this section, the possible explanations for the findings regarding differences by age and sex, especially the fluctuations in homicide rates and the Rate Ratio (RR) between the burden of rural and urban homicide, will be analyzed.

### 4.1. Possible Explanations

Regarding the distribution by sex, homicide rates for men were much higher than those for women, and this difference is considerably higher than in other countries [7]. It can also be observed that the fluctuations in homicide rates for both sexes match, which is consistent with the previous findings of other global studies [7, 32].

That said, with respect to the behaviour of the homicide rate in Colombia for the period 1992-2015, this work identified in general four critical inflexion points in which homicide rates for rural and urban areas and for both sexes are reasonably similar: 1992, 1997, 2002, and 2005/2006.

1992, the first year of our study, has been reported by other trends studies as one of the spikes of lethal violence in Colombia in recent decades [3, 15, 33–35]. The results disaggregated by area show, from that year on, a statistically significant fall in urban homicide rates until 1997, although in this same period there are no significant changes in rural homicide rates. This reflects the urban root of this peak in 1992, which, according to the same sources, arises from a gradual increase in the homicide rate since the mid-1980s, coincides with the growing activity of urban drug cartels, and progressively decreases only in cities.

Then, in 1997-2002 there was an increase in homicidal violence that was pronounced and statistically significant in rural but not in urban settings, which accounts for the predominance of rural violence at this peak. At this stage, the use of massacres as a mechanism of territorial control, the conversion of the civilian population into a target by the participants and a greater number of deaths in combat between irregular groups and the Security Forces are documented [31, 36, 37]. Additionally, this was the period of the paramilitaries' “challenge to the guerrilla order” (1996-2002) [38]. A study for the period 1998-2003 shows that, while the conflict was most intense mainly in the areas where the first guerrilla nuclei were established, more than a third of the armed actions occurred in very rural communities [39]. The increase in demand for coca leaf in Colombia during this period generated an increase in self-employed income and an increase in work opportunities for the inhabitants of coca-growing areas, which coincided with the escalation of violence [14]. Contrary to what might be expected, the rural municipalities where a higher intensity of violence was recorded at the time were characterized by being areas of commercial agricultural activities and having low poverty rates [18]. These zones were also marked by having unequal distributions of income, having uncontrolled local booms, and evidencing poor infrastructure and an insufficient presence of state institutions [40].

As of 2002, the decline in rural and urban homicide rates can be attributed to a large extent to the increase in the operational capacity of the Security Forces, which forced the irregular groups to retreat and therefore reduced the number of victims. The demobilization of the self-defence groups also contributed to this reduction [9]. Therefore, from 2002 until 2005 in men and 2006 in women, there is a very pronounced reduction of homicides in rural and urban areas. From this point, the fall remains constant but slows down during the last decade of the period studied. There is a slight peak in urban homicide rates, particularly in 2009, which could be related to a change in the tactics implemented by the FARC, which sought to multiply the scenarios of confrontation and weaken the army morally and physically in different parts of the country [18], as well as struggles for power in other armed illegal urban organizations as their leaders were captured [20].

Regarding the relationship between rural and urban homicide according to the Rate Ratio, in almost the whole period we found higher mortality rates in rural than in urban areas, different from most evidence around the world. As previously shown dynamics of internal armed conflict in rural settings partially explain this uncommon divergence. However, the broadening in the gap in homicide rates among rural and urban settings was not only probably due to intensification of violence in rural settings but also for interventions addressed to solely reduce urban violence in major Colombian cities. Medellin for example experienced a striking reduction in homicide rates between 1993 and 2015 (400 to 20 homicides by 100,000 population) [41]. In most recent years this result was attributed to the so-called “hot spots policing” which was implemented in 2008 in Bogota and Medellin [42] and in 2010 in other major cities [43]. This strategy involves the targeting of resources and activities to those places where crime is most concentrated with intensive deployment of police force, social prevention activities, and improvement in urban environments. Nevertheless another study did not find significant evidence to confirm the actual effect of hot spot policing since researchers cannot control spillover effects [44]. On the other hand, organizations like Humans Right Watch have coined the term “guns covenant” referring to sophisticated agreements among criminal organizations to self-restrict themselves and reduce the notoriety of the their crimes, lessening their visibility in front of authorities and public opinion and allowing them to peacefully manage their illegal business [41]. All this could also contribute to the deepening of the gap between urban and rural settings. In addition, in recent years RRs in men show a progressive predominance of urban homicides, at the expense of a more accented and continuous reduction in rural homicide rates. According to this, it could be hypothesized that this divergence will continue to increase—with a potential stagnation in urban homicide—constituting a new challenge for the future. In addition, by disaggregating the RR by age, it is striking that while there was a higher burden of rural violence in men over 25 years of age during most of the period studied and until 2015 (as in women aged 15-64), we found that the highest urban homicide rates during practically the whole period studied were for the youngest men (15-24). Contrary to what has been proposed by initial studies on the role of unfavourable sociodemographic conditions in youth violence [35], it has been identified that the incorporation of young people into organized crime is also motivated by the social status acquired by joining a criminal gang or receiving training by armed groups [45]. Recent research indicates that youth violence in urban contexts is associated mainly with organized crime and less with the armed conflict [46]. In Colombia, the severity of juvenile violence rates varies from one city to another, depending on the intensity of the confrontations between the different groups existing in each area and their degree of professionalization. In this vein, as long as the risk factors are not addressed, such as the lack of access to education, the low rates of urban labour participation in young people, and the inability of the family unit to provide a safe environment, violent youth gangs have a high level of probability of progressing towards organized crime groups, which implies a clear challenge for postconflict Colombian society [46].

### 4.2. Implications

As has been previously demonstrated, institutional weaknesses contribute more to Colombia's high rates of violence than social inequality does, contrary to studies in societies with moderate or low rates of violence—essentially urban—that have strong regulations and social control systems that maintain cohesion. In those settings, a direct relationship between violence and inequality has been found [47]. It could be speculated from our findings that as the deterrent power of the state becomes more effective and the peace and demobilization processes with the remaining armed groups' progress [20], reducing social inequality could become a more important factor in continuing the reduction in homicide rates. The fact that the predominance of urban homicides in young men coincides with the slowdown in the reduction of homicides at the end of the period cannot be underestimated. In fact, it has been found that economic growth, inequality, poverty, and human capital, as well as different levels of development, also influence the levels of urban violence in Colombia [48].

According to this study's findings, it is important to prioritize a greater understanding of urban youth violence in Colombia by gathering more evidence and reviewing and updating the time series on the subject in the country [48–55]. It is also important to emphasize that strategies which have been shown to be effective in reducing homicides in localized areas of large cities [55–58] should be more widely studied and implemented.

A key challenge for the Colombian state is how to find appropriate legal alternatives to issues such as the production of illicit crops (in rural areas) and the commercialization of drugs in urban areas, when these are seen as valid and profitable career choices as opposed to the poorly paid work of raising crops that take time to bring a financial return in rural areas and low-paid jobs in urban areas.

Finally, as there are no registries of potential determinants of violence (deterrence or socioeconomic variables for instance) separately for urban and rural settings, comparative analyses of violence at that level remain being speculative. In the same line, while researchers do not have baseline variables to perform ex-post-studies in times of noticeable reduction in violence (e.g., early 90's in rural settings, or the beginning of this century, or during recent years at different negotiations with armed groups), studies as this one will not be able to provide reasonable certainty about the factors or strategies for a sustained reduction in rural and urban lethal violence.

### 4.3. Conclusions

The results of this study show that unlike other countries, the variations in homicide rates in Colombia are more pronounced in rural areas than in urban areas, which coincides with periods of worsening violence in the regions most affected by the armed conflict. In view of the fact that in the last years of this study urban lethal violence is on a par with, and sometimes exceeds, rural violence, in a postconflict scenario it is likely that the phenomenon of homicide, especially by younger men, will become more important in cities.

With the increasing ability of the state to provide an effective deterrent and the reduction of rural violence, decision makers must fix their sights on the implementation of measures that reduce social inequalities with emphasis on the most disadvantaged youth population, especially in the cities, in order to be able to maintain the decreasing trend of homicide rates in Colombia. This may imply evaluating and intervening in other variables related to poverty and inequality if we want to continue with the reduction of homicidal violence. It is proposed that future studies evaluate how aspects directly related to living conditions or the development of rural areas could significantly affect the behaviour of urban homicide rates in the postconflict context.

## Figures and Tables

**Table 1:** Counts (deaths and population), age standardized homicide rates (ASHR), and rate ratios (RR) of rural/urban (reference) homicide, among Colombian men and women aged 15-64, for the whole period (1998-2015), separately by age groups and urban/rural area.

**Sex**	**Variable**	**Category**		**Counts**				**Age-standardized ** **Homicide rate**		**Rate Ratio (RR) ** **rural/urban**	
				**Population ** *∗*		**Homicides**					
				#	%	#	%	ASHR	SE	RR	SE
											
**MEN**	Area of residence		Urban	226.998	73%	330.602	71%	91,49	0,00	**Ref**	
			Rural	82.142	27%	134.700	29%	107,06	0,16	-	
										
	Age group	Adolescent and youth	(15-24 years)	93.632	30%	158.023	34%	170,44	0,43	0,87	0,01
		Young adults	(25-44 years)	138.596	45%	246.482	53%	176,27	0,36	1,26	0,00
		Middle aged adults	(45-64 years)	76.912	25%	60.797	13%	77,92	0,32	1,47	0,01
										
	**TOTAL**			**309.140**	**100**%	**465.302**	**100**%	**95,37**	**0,14**	**1,15**	**0,01**

**WOMEN**	Area of residence		Urban	250.711	78%	25.953	70%	6,62	0,29	**Ref**	
			Rural	72.060	22%	11.002	30%	9,78	0,04	-	
										
	Age group	Adolescent and youth	(15-24 years)	92.524	29%	12.564	34%	13,61	0,12	1,36	0,02
		Young adults	(25-44 years)	146.513	45%	18.681	51%	12,70	0,09	1,41	0,02
		Middle aged adults	(45-64 years)	83.733	26%	5.710	15%	6,73	0,09	1,66	0,03
										
	**TOTAL**			**322.770**	**100**%	**36.955**	**100**%	**7,31**	**0,04**	**1,40**	**0,02**

Conventions:

#: population and homicide counts; population counts are understood as population-year.

%: percentage of population and homicides, respectively.

ASHR: homicide rate per 100,000 population, standardized by age (WHO standard population).

SE: standard error of homicide rate per 100,000 population, standardized by age (WHO standard population).

Rate ratios (RR): rural/urban (reference); estimates were calculated using Poisson regression models using region, sex, and 5-year age group as covariates.

## Data Availability

This study is based on consolidated yearly datasets of anonymized individual deaths and on aggregated population data. All information is publicly provided by DANE in its webpage. It is also available from the corresponding author upon request.
